# Dual function of partitioning-defective 3 in the regulation of YAP phosphorylation and activation

**DOI:** 10.1038/celldisc.2016.21

**Published:** 2016-07-05

**Authors:** Peng Zhang, Shuting Wang, Sai Wang, Jing Qiao, Lei Zhang, Zhe Zhang, Zhengjun Chen

**Affiliations:** 1 Key Laboratory of Systems Biology, Institute of Biochemistry and Cell Biology, Shanghai Institute for Biological Sciences, Chinese Academy of Sciences, Shanghai, China; 2 University of Chinese Academy of Sciences, Beijing, China; 3 State Key Laboratory of Cell Biology, Institute of Biochemistry and Cell Biology, Shanghai Institute for Biological Sciences, Chinese Academy of Sciences, Shanghai, China; 4 School of Life Science and Technology, ShanghaiTech University, Shanghai, China

**Keywords:** LATS1, Par3, PP1A, YAP

## Abstract

Partitioning-defective 3 (Par3), a key component of the evolutionarily conserved polarity PAR complex (Par3/Par6/aPKC), controls cell polarity and contributes to cell migration, proliferation and tumor development. Emerging evidence indicates that cell polarity proteins function as upstream modulators that regulate the Hippo pathway. However, little is known about Par3’s involvement in the Hippo pathway. Here, we find Par3 and YAP dynamically co-localize in different subcellular compartments; that is, the membrane, cytoplasm and nucleus, in a cell-density-dependent manner. Interestingly, Par3 knockdown promotes YAP phosphorylation, leading to a significant impairment of YAP nuclear translocation at low cell density, but not at high density, in MDCK cells. Furthermore, via its third PDZ domain, Par3 directly binds to the PDZ-binding motif of YAP. The interaction is required for regulating YAP phosphorylation and nuclear localization. Mechanistically, Par3, as a scaffold protein, associates with LATS1 and protein phosphatase 1, α subunit (PP1A) in the cytoplasm and nucleus. Par3 promotes the dephosphorylation of LATS1 and YAP, thus enhancing YAP activation and cell proliferation. Strikingly, we also find that under the condition of PP1A knockdown, Par3 expression promotes YAP hyperphosphorylation, leading to the suppression of YAP activity and its downstream targets. Par3 expression results in differential effects on YAP phosphorylation and activation in different tumor cell lines. These findings indicate that Par3 may have a dual role in regulating the activation of the Hippo pathway, in a manner possibly dependent on cellular context or cell type in response to cell–cell contact and cell polarity signals.

## Introduction

Cell polarity determines spatial differences in cell structures and shapes and is fundamental for tissue functions. The structural and functional polarity axis (apical–basal polarity) of epithelial cells is characterized and maintained by three well-conserved polarity complexes: Par, Crumbs and SCRIB [1]. The Par complex, that is, the Par3/Par6/atypical protein kinase C (aPKC) complex, is an evolutionarily conserved regulator of the initiation of cell polarity in *Caenorhabditis elegans* and *Drosophila* embryos and the asymmetric cell division of *Drosophila* neuroblasts [2–4]. Partitioning-defective 3 (Par3), a signaling scaffold protein in the Par3/Par6/aPKC complex, contains a conserved N-terminal domain, three PSD-95/Discs-large/ZO-1 (PDZ) domains and a C-terminal region including the aPKC-binding-motif and coiled-coil domain. These domains mediate protein–protein interactions and have critical roles in Par3’s regulation of various modes of polarization during neuronal development, migration and tight junction (TJ) formation in vertebrate cell polarity [5–7]. Par3 knockdown in MDCK cells severely disrupts TJ formation and cells fail to form normal cysts [8]. During the formation of the PAR complex, PAR3 interacts with the Rac-specific guanine nucleotide exchange factor Tiam1, which binds to integrins through talin, and regulates Rac1 activity and adhesion turnover for polarized migration [9, 10]. aPKC and/or Par3 control spindle orientation and cell fate decisions in the developing mammary gland, the epidermis and in radial glial cells, similar to what is observed in *C. elegans* and *Drosophila* [11]. The proper localization of Par3 is required for establishing neuronal polarity and SC myelination [12, 13].

In addition to its roles in cell polarity, Par3 is involved in other cellular functions and tumor development. For instance, γ-irradiation-induced Par3 translocates into the nucleus, where it binds to Ku70 and Ku80, the regulatory subunits of the DNA-dependent protein kinase, thereby affecting double-strand break repair [14]. Hepatocyte growth factor (HGF) treatment induces Par3 nuclear translocation in MDCK cells, which has been proposed to be an early event during HGF-induced endothelial–mesothelial transition [15]. Par3 has been reported to bind to the high-risk HPVE6 protein, leading to its cellular mislocalization in a PDZ-dependent manner [16]. Wang *et al.* [17] have shown that, upon stimulation by multiple growth factors, Par3 is tyrosine-phosphorylated by Src kinases, such as c-Src and c-Yes, and this phosphorylation is required for its dissociation from LIM kinase 2, regulation of cofilin activation and assembly of epithelial TJs. Par3 phosphorylation mediated by the serine/threonine kinases Par1 has been implicated in the disruption of epithelial apical–basal polarity and cyst formation [18]. Par3 interacts with dynein light intermediate chain 2 (LIC2), regulating microtubule dynamics at cell–cell contacts and the proper positioning of the centrosome at the cell center [19]. These data have indicated that Par3 subcellular distribution and phosphorylation, controlled by different cell signals, is essential for a variety of its functional outcomes.

Several recent studies have implicated Par3 in the development of various tumor models. Inhibiting Par3 causes a loss of cell polarity and promotes tumorigenesis and metastasis of breast cancer, pancreatic cancer and lung squamous cell carcinoma [20, 21]. In a mouse model, Par3 has been demonstrated to have a tumor type-dependent function in chemical-induced skin tumorigenesis. Par3 deficiency results in reduced papilloma formation and growth. However, Par3-deficient mice are predisposed to keratoacanthoma formation [22]. The increased expression of Par-3 in primary hepatocellular carcinoma tissues is associated with poor 5-year overall survival rates [23]. Thus, these findings imply that the dual function of Par3 in cell proliferation and tumorigenesis is possibly dependent on physiological cellular contexts or cell types. However, the molecular mechanism underlying its contradictory roles in cell proliferation and tumorigenesis remain poorly understood.

The components of TJs, adherens junctions and apical–basal polarity protein complexes including α-catenin, E-cadherin, NF2 (Merlin), Amot (Angiomotin) and Crb (Crumbs) have been identified as important upstream regulators of the activation of the Hippo/YAP (Yes-associated protein) pathway [24–26]. The key components of the signaling pathway consist of two serine/threonine kinases, Mst1/2 (Hippo) and large tumor suppressor kinase 1/2 (LATS1/2) (Warts), which negatively regulate the transcriptional cofactor YAP/TAZ by phosphorylation, thus causing its retention in the cytoplasm [27]. In the absence of Hippo upstream signaling, hypophosphorylated YAP translocates to the nucleus, where it binds to DNA with sequence-specific transcription factor TEAD and triggers the transcription of target genes, including *Ankrd1, Ctgf, Cyr61, cyclin E* and *Diap*, whose protein products encourage cell proliferation and prevent apoptosis, respectively [28]. In addition to YAP regulating proliferation and interacting with adherens junction- and apical domain-associated proteins, overexpression of aPKC or depletion of Scrib or Lgl also results Yki activation [26]. However, the involvement of the Par complex in the regulation of the Hippo/YAP pathway has been investigated less thoroughly. Here, we report our findings on the roles of Par3 in the regulation of the Hippo/YAP signaling pathway.

## Results

### Par3 is involved in YAP subcellular translocation in MDCK cells

**Figure 1:**
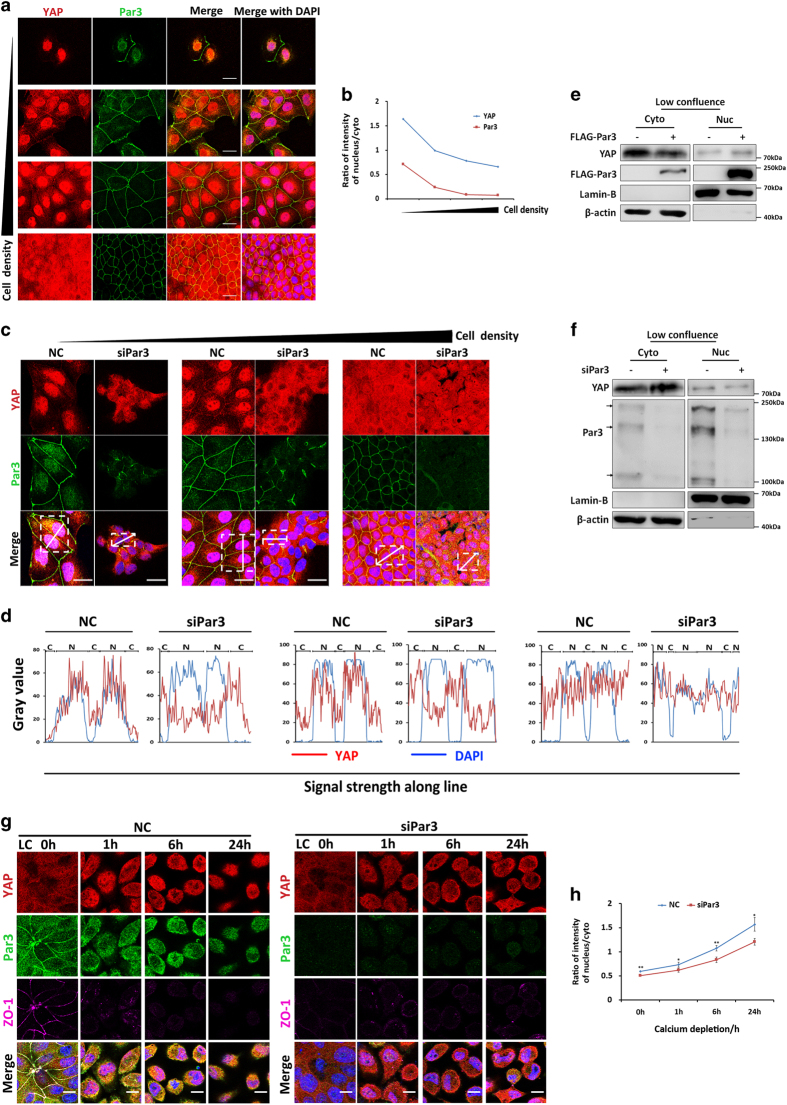
Par3 expression affects YAP subcellular translocation in MDCK cells. (**a**) Distribution and co-localization of YAP (red), Par3 (green), a merged image with only YAP and Par3 and a merged image with 4′, 6-diamidino-2-phenylindole (blue) of MDCK II cells at different cell densities. Scale bar: 25 μm. (**b**) Ratios of the percentages of nuclear and cytoplasmic YAP and Par3 at different cell densities are shown. Pictures were analyzed with a Columbus Image Data Storage and Analysis System. (**c**) Representative images of YAP translocation into the cytoplasm at low cell density when Par3 was knocked down but not at high cell density. MDCK II cells were transfected with siRNA for Par3 at different cell densities. YAP (red), Par3 (green) and a merged image. Scale bar: 25 μm. All pictures were taken with a Leica TCS SP5 microscope. (**d**) The areas representing the cytosolic or nuclear fraction are indicated with bars at the top of each histogram. N: nucleus; C: cytoplasm. The histogram analysis of YAP and Par3 signal intensity in subcellular distributions was performed with the ‘Plot profile’ in ImageJ software. The analyzed area is indicated by the white straight line in the overview. YAP signal intensity distribution (red line), nucleus signal intensity distribution (blue line). (**e**, **f**) YAP translocated into the nucleus with Par3 overexpression, and YAP nuclear localization was inhibited when Par3 was knocked down. A cell fraction assay was performed after 293T cells were transfected with Flag-Par3 or MDCK II cells were transfected with siRNA for Par3 for 2 days at low cell density. Lamin-B is the nuclear marker and β-actin is the cytoplasmic marker. (**g**) Par3 knockdown reduced YAP translocation to the nucleus in the Ca^2+^ off switch system. Representative images of YAP translocation into the nucleus at high cell density when Ca^2+^ was depleted are shown. After 2 days, while MDCK II cells were transfected with siRNA for Par3, MDCK II cells were cultured with Ca^2+^-free medium for the indicated time course. YAP (red), Par3 (green) and ZO-1 (purple). Scale bar: 25 μm. (**h**) Ratios of the percentages of nuclear and cytoplasmic YAP and Par3 at different time points. Pictures were analyzed with a Columbus Image Data Storage and Analysis System. **P<0.01, *P<0.05.

A previous study from our lab has reported that Par3 translocates from the membrane and the cytoplasm into the nucleus in γ-irradiated MDCK cells [14]. Indeed, Par3 nuclear localization was clearly detected at low cell density in MDCK cells (Figure 1a) and HeLa cells [14]. Its nuclear translocation increased as cell density decreased (Figure 1a), which was quantified by Columbus Image Analysis System (Figure 1b). It is well known that cell density regulates the activity of the Hippo pathway and TAZ/YAP localization [27]. Consistently with this finding, YAP translocation from the nucleus to the cytoplasm and cell membrane was regulated by increased cell density (Figure 1a–c). Interestingly, we observed similar dynamic behaviors in the cellular distribution of both Par3 and YAP in response to cell-density alteration (Figure 1b). Further analysis revealed subcellular co-localization of Par3 with YAP in the nucleus, cytoplasm and membrane in sparse and confluent cell cultures, as shown in Figure 1a and c. At low cell density, Par3 primarily co-localized with YAP in the nucleus and the cytosol. Co-localization of Par3 and YAP occurred on the membranes when the TJs were completely established at high cell density. Collectively, these results demonstrated that subcellular co-localization of Par3 and YAP was regulated by cell density.

Thus, we sought to investigate whether Par3 is involved in YAP activation. Immunofluorescence analysis exhibited a dramatic reduction of nuclear YAP in RNAi-mediated Par3-knockdown MDCK cells compared with the control cells; that is, in particular, in sparsely grown cultures but not at a high cell density (Figure 1c). These image signals were quantified by the histogram analysis (Figure 1d), which clearly proved that Par3-knockdown inhibits YAP nuclear localization at low cell density (Figure 1d). Par3 knockdown efficiencies at different densities were determined by western blotting (Supplementary Figure S1A). A subcellular fractionation assay demonstrated that when Par3 was overexpressed in 293T cells, more YAP was detected in the nucleus and at relatively low levels in the cytosol at low cell density (Figure 1e), whereas no obvious translocation was observed at high cell density (data not shown). In contrast, Par3 knockdown in MDCK cells resulted in enriched YAP in the cytoplasmic fraction and reduced YAP in the nucleus at low cell density (Figure 1f), but it was not changed at high cell density (data not shown). In epithelial cells, the establishment of adherens junctions, TJs and apical–basal polarity is Ca^2+^-dependent, and growth in Ca^2+^-free media (Ca^2+^ depletion) results in disruption of these cellular junctions in cultured cells [29]. Disrupting cell junctions by Ca^2+^ depletion and a dramatic shift of TAZ/YAP localization from the cytoplasm to the nucleus have been observed in Eph4 cells [24]. We used this system to determine the influence on YAP translocation in the nucleus and cytoplasm by Par3 knockdown with immunofluorescence analysis. We observed a dramatic shift of YAP nucleus/cytoplasm shuttling of MDCK cells during the time course of Ca^2+^ depletion and recovery (Figure 1g left panel and Supplementary Figure S1B left panel). In addition, a similar shift of Par3 was detected in those cells (Figure 1g left panel and Supplementary Figure S1B left panel). Quantitative analysis revealed an increasing dynamic nucleus/cytoplasm ratio of YAP from 0.6 to 1.5 at different time points of Ca^2+^ depletion; however, the YAP nucleus/cytoplasm ratio significantly decreased to 0.5–1 when Par3 was knocked down (Figure 1g right panel and Figure 1h). In a Ca^2+^ recovery system, YAP showed a fast translocation from the nucleus to the cytoplasm and plasma membrane (Supplementary Figure S1B left panel), and the ratio exhibited a very rapidly declining curve from 3 to 0.5 in the MDCK control cells (Supplementary Figure S1C). However, compared with the control cells, the ratio in Par3 knockdown cells had a flatter downward curve from 2.3 to 0.9 (Supplementary Figure S1B right panel and Supplementary Figure S1C). Together, these data demonstrated that Par3 is involved in YAP nucleus/cytoplasm shuttling.

### Par3 binds to the YAP PDZ-binding motif (PDZM) via its PDZ3 domain

**Figure 2:**
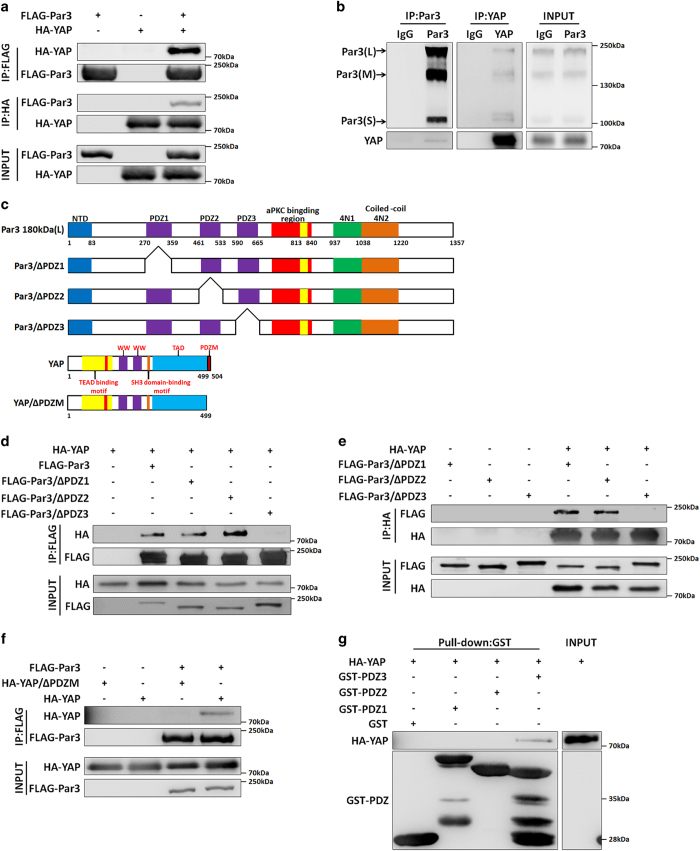
Par3 binds to the YAP PDZ-binding motif (PDZM) via its PDZ3 domain. (**a**) Par3 interacted with YAP. 293T cells were transiently transfected with the FLAG-Par3 and HA-YAP as indicated; co-immunoprecipitation (co-IP) and reverse-IP with anti-FLAG/HA antibodies were performed, and the cells were harvested for western blot analysis. (**b**) Endogenous Par3 bound to YAP in MDCK II cells. MDCK II cells were harvested and co-immunoprecipitated and reverse-immunoprecipitated with anti-Par3/YAP antibodies. (**c**) Modular structures of the three PDZ domain deletion mutations of Par3 and YAP. (**d, e**) Deletion of the Par3 PDZ3 eliminated its interaction with YAP. 293T cells were transfected with FLAG-Par3, FLAG-ΔPDZ1/2/3 mutants of Par3 and HA-YAP; co-IPs and reverse-IPs were conducted with anti-FLAG/HA antibodies. (**f**) Deletion of the PDZ motif of YAP also abrogated binding to Par3. 293T cells were transfected with FLAG-Par3, HA-YAP and HA-YAP/ΔPDZM; co-IPs were performed with anti-FLAG antibodies. (**g**) PDZ3 of Par3 was essential for the interaction, as determined by pull-down assay. 293T cells, which were transfected with HA-YAP, were lysed and incubated with glutathione-S-transferase (GST) and GST-tagged PDZ1/2/3 domains of Par3 bound to glutathione beads.

The dynamic co-localization of Par3 and YAP induced by different cell densities raised an interesting question; that is, whether Par3 is associated with YAP. To address this issue, we performed co-immunoprecipitation assays in HEK293T cells wherein FLAG-tagged Par3 and hemagglutinin (HA)-tagged YAP were transfected either alone or together. As shown in Figure 2a, FLAG-Par3 was detected in the HA-YAP immune complex, whereas HA-YAP was found in the FLAG-Par3 immune complex. However, we did not detect any association of YAP with aPKC and Par6, the other two components of the Par complex (Supplementary Figure S2A). We further examined whether endogenous Par3 interacts with YAP in MDCK cells. Endogenous Par3 co-immunoprecipitated with endogenous YAP, an association that did not occur with the control IgG. Reciprocally, endogenous YAP co-precipitated with three endogenous isoforms of Par3 (Figure 2b), which contain all three PDZ domains [30]. These results indicated that Par3 forms a physiological complex with YAP in cells.

To map the interacting domains between Par3 and YAP, we generated Par3 deletion mutants lacking its three PDZ domains, denoted here as Par3/∆PDZ1, Par3/∆PDZ2, Par3/∆PDZ3, and a YAP deletion mutant lacking the PDZ-binding motif (YAP/∆PDZM) [31] (Figure 2c). Co-immunoprecipitations and reverse-immunoprecipitations conducted in 293T cells (Figure 2d and e) showed that deletion of Par3 PDZ3 but not PDZ1 or PDZ2 disrupted Par3–YAP interaction. In addition, as expected, YAP/∆PDZM was not able to interact with Par3/wt (Figure 2f). To refine the binding site, an *in vitro* pull-down assay with glutathione-S-transferase fusion proteins of Par3 PDZ1/2/3 domains verified that Par3 PDZ3 specifically and directly binds to YAP (Figure 2g). These data indicated that Par3 and YAP form a novel complex mediated by the PDZ-PDZM binding module.

### Par3 promotes YAP dephosphorylation and activation, regulating YAP target gene expression and cell proliferation

**Figure 3:**
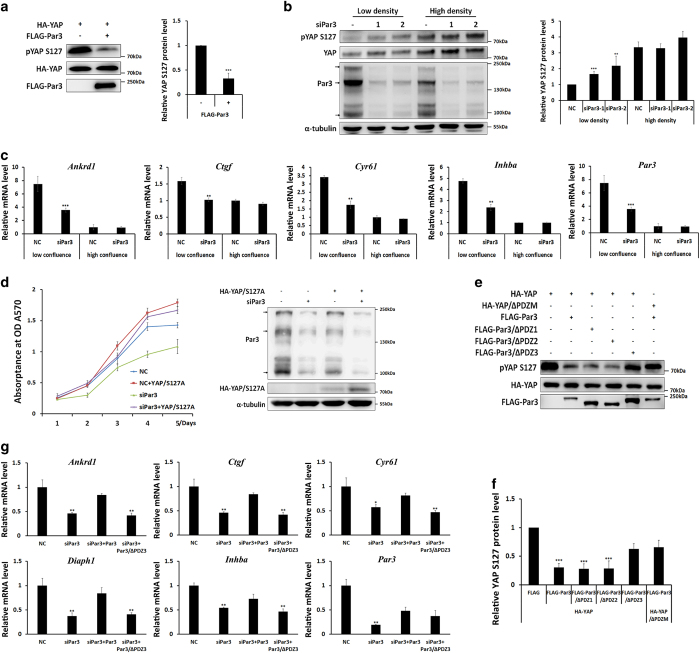
Par3 promotes YAP dephosphorylation and activation, regulating YAP target gene expression and cell proliferation. (**a**) Par3 reduced YAP phosphorylation at Ser127. 293T cells were transfected with FLAG-Par3 and HA-YAP, and the pYAP Ser127 site was detected. The data are representative of three independent experiments, ***P<0.001. (**b**) Par3 knockdown increased YAP phosphorylation at low cell density but not at high cell density. MDCK II cells were transfected with two different siRNAs for Par3 at low and high cell densities, and total YAP and pYAP Ser127 sites were detected. The data are representative of three independent experiments, ***P<0.001, **P<0.01. (**c**) Par3 knockdown inhibited YAP target genes *Ankrd1, Ctgf, Cyr61* and *Inhba* at low cell density but not at high cell density. RT-qPCR was performed in MDCK II cells transfected with siRNA for Par3 at low and high cell densities. (**d**) Par3 knockdown inhibited cell proliferation, whereas overexpression of YAP active forms and YAP S127A rescued the inhibition by Par3 knockdown in MDCK II cells. An MTT assay was performed in MDCK II cells, and Par3 and HA-YAP and YAP/S127A expression levels were determined by western blotting. (**e**, **f**) Deleted PDZ3 domain of Par3 could not reduce YAP phosphorylation and deleted PDZ motif of YAP could not be regulated by Par3. 293T cells were transfected with indicated plasmids. (**g**) The inhibition of YAP target genes (*Ankrd1, Ctgf, Cyr61, Diaph1* and *Inhba*) by Par3 knockdown at low density could be partially rescued by Par3, but not by Par3 ΔPDZ3. RT-qPCR was performed in MDCK II cells transfected with siRNA for Par3 at low density.

To examine the functional significance of the interaction, we considered whether Par3 could influence YAP phosphorylation at serine 127 (Ser127), which is crucial for its localization and activity [32]. We observed that Par3 overexpression resulted in a significant reduction of YAP phosphorylation (Figure 3a). Next, we determined whether Par6 and aPKC regulate YAP activation, because Par6 and aPKC form a polarity protein complex with Par3 to regulate various cell polarization events. Expression of either aPKC or Par6 had no influence on YAP phosphorylation (Supplementary Figure S2A). These results suggested that Par3 might regulate YAP phosphorylation independently of the Par complex. Next, Par3 was knocked down using siRNAs in MDCK cells. Interestingly, we found that, at low cell density, Par3 knockdown led to a clear increase in endogenous YAP phosphorylation, whereas Par3 knockdown had no obvious effect on YAP phosphorylation at high cell density (Figure 3b). Similar results were obtained in the MDCK/Par3Ri cells in which Par3 was stably knocked down (Supplementary Figure S2B). These findings suggested a critical role for Par3 in regulating YAP phosphorylation in a cell density-dependent or cell–cell contact-dependent manner. YAP dephosphorylation is important for its co-transcriptional activity, thereby modulating its target gene expression and cell proliferation [32]. Next, we determined the functional consequences of Par3 on YAP’s target gene expression by using a real-time PCR approach. As expected, knockdown of Par3 significantly decreased the mRNA expression levels of *Ankrd1, Ctgf, Cyr61* and *Inhba* at low cell density, whereas no significant changes were detected at high cell density (Figure 3c). Furthermore, a thiazolyl blue tetrazolium bromide (MTT) assay demonstrated that Par3 knockdown significantly inhibited cell growth, which could be rescued by overexpression of YAP/S127A, a constitutively active mutant (Figure 3d).

Next, we asked whether Par3–YAP binding is involved in YAP phosphorylation. To clarify the issue, co-overexpression experiments were performed with Par3 and its PDZ deletion mutants (Par3/∆PDZ1, 2, 3), YAP and its YAP/∆PDZM mutant. As shown in Figure 3e, expression of Par3/wt and its mutants in 239 T cells, that is, Par3/∆PDZ1 or Par3/∆PDZ2, notably reduced YAP phosphorylation, but the YAP-unbound Par3/∆PDZ3 mutant did not (Figure 3e). Furthermore, Par3 overexpression had no effect on YAP/∆PDZM phosphorylation in comparison with YAP/wt (Figure 3e). These results were quantified by image density analysis (Figure 3f), which proved the importance of Par3–YAP binding for YAP dephosphorylation modulated by Par3. In addition, we also examined the requirement of the interaction between Par3 and YAP for YAP target gene expression. As shown in Figure 3g, indeed, the reduction of all tested target genes by Par3 knockdown could be significantly rescued by overexpressing Par3/wt but not Par3/∆PDZ3 (Figure 3g). The above data demonstrated that Par3–YAP binding is required for Par3-induced YAP dephosphorylation and activation. Together, these findings indicated a positive regulatory role for Par3 in stimulating YAP activity and YAP-mediated cell proliferation.

### Par3 interacts with LATS1/2, YAP and PP1A and promotes dephosphorylation of LATS1 and YAP

**Figure 4:**
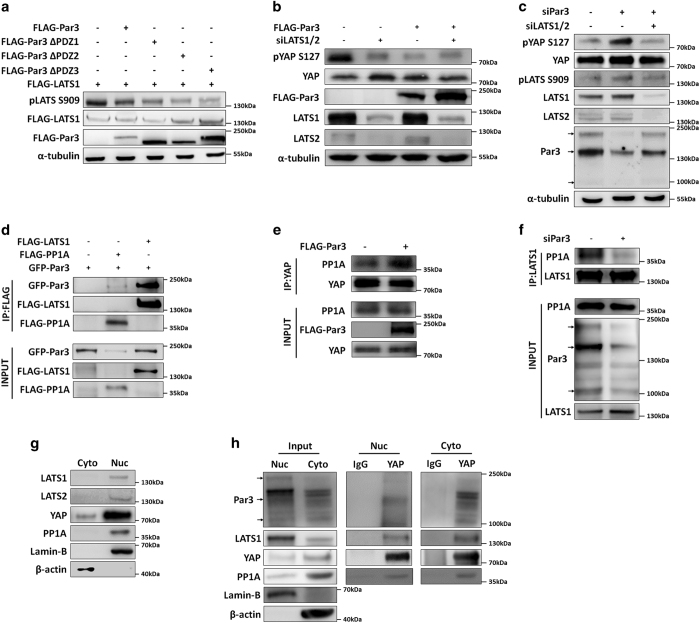
Par3 interacts with LATS1/2, YAP and PP1A and promotes dephosphorylation of LATS1 and YAP. (**a**) Overexpression of Par3 and PDZ deletion mutants decreased LATS1 phosphorylation at Ser909. 293T cells were transfected with the indicated plasmids, and pLATS Ser909 was determined by western blotting. (**b**) The reduction of YAP phosphorylation by Par3 overexpression was weakened when LATS1/2 was knocked down. 293T cells were transfected with FLAG-Par3 and siRNA for LATS1/2; western blot analysis was performed as indicated. (**c**) The increment of YAP phosphorylation by Par3 knockdown was attenuated by LATS1/2 knockdown. MDCK II cells were transfected with siRNA for Par3 or LATS1/2; western blot analysis was performed as indicated. (**d**) Par3 interacted with LATS1 and PP1A. Co-immunoprecipitation was conducted with anti-FLAG antibodies in 293T cells, which were transfected with GFP-Par3, FLAG-LATS1 and FLAG-PP1A. (**e**) Par3 promoted the interaction of YAP and PP1A. IP was conducted with anti-YAP antibodies in 293T cells transfected with FLAG-Par3, and endogenous PP1A was subjected to western blot analysis. (**f**) Par3 knockdown reduced the interaction of LATS1 and PP1A. IP was performed with anti-LATS1 antibodies in 293T cells transfected with siPar3, and western blot analysis was performed as indicated. All experiments were performed at low cell density. (**g**) LATS1 and PP1A mainly localized in the nucleus at a low cell density. Cell fractionations were performed with MDCK II cells at low cell density. Western blot analysis was performed as indicated. (**h**) YAP interacts with Par3, LATS1 and PP1A in the nucleus and cytoplasm. IP with anti-YAP antibodies was conducted with the nuclear and cytoplasmic fractions of MDCK II cells at low cell density. Western blot analysis was performed as indicated.

YAP phosphorylation at Ser127 is mediated by LATS1/2 activity [33]. LATS1/2 phosphorylation at tyrosine 1079 (Tyr1079) is controlled by MST1/2, thereby leading to LATS1/2 autophosphorylation at serine 909 (Ser909) [34]. To elucidate the molecular mechanisms underlying Par3-modulated YAP dephosphorylation, we first asked whether Par3 regulates LATS phosphorylation and activation. We performed co-transfection experiments and observed that LATS1 phosphorylation at Ser909 was decreased by overexpression of Par3/wt and its PDZ deletion mutants (Figure 4a), indicating that none of these PDZ domains were involved in Par3-mediated LATS1 dephosphorylation. As expected, in the case of LATS1/2 knockdown, Par3/wt expression did not further decrease YAP phosphorylation (Figure 4b). Par3 knockdown clearly increased phosphorylation of LATS1 at Ser909 and YAP phosphorylation (Figure 4c). However, simultaneous knockdown of Par3 and LATS1/2 caused dephosphorylation of YAP (Figure 4c), indicating that Par3 might inactivate LATS1, thereby inhibiting YAP phosphorylation. These data revealed that LATS1/2 is required for the regulation of YAP phosphorylation modulated by Par3.

Next, we asked how Par3 dephosphorylates LATS1 and YAP. Protein phosphatase 1 (PP1) has been reported to bind to Par3 and regulate Par3 phosphorylation [35]. PP1A, the catalytic subunit of PP1, has also been reported to be required for YAP dephosphorylation [36]; however, the underlying mechanism is unclear. In fact, in addition to Par3–YAP interaction, we also detected that Par3 was strongly associated with LATS1 and PP1A (Figure 4d). Interestingly, Par3 overexpression promoted association of YAP with PP1A (Figure 4e). Furthermore, Par3 knockdown reduced the interaction between LATS1 and PP1A (Figure 4f). Together, our results indicated that Par3 might act as a signaling platform coordinating the association with YAP, PP1A and LATS1, thereby downregulating the Hippo signaling pathway and activating YAP.

YAP phosphorylation by LATS1/2 has been proposed to occur in the cytoplasm or at the plasma membrane [37, 38]. Recently, phosphorylated LATS1 has been reported to accumulate almost exclusively in the nucleus in different cell lines [39]. PP1A has also been reported to be localized in the nucleus [40]. Therefore, we sought to determine whether Par3 is associated with YAP, LATS1/2 and PP1A in the nucleus, because Par3 clearly co-localized with YAP in the nucleus (Figure 1a). Cell fractionation analysis revealed that LATS1/2, PP1A and YAP were detected in the nuclear fraction at low cell density (Figure 4g). Consistent with these observations, immune-staining analysis revealed that LATS1 and PP1A were localized in the nucleus at low cell density, but translocated to the cytoplasm in high-density MDCK cells (Supplementary Figure S3A and D). Interestingly, strong signals for pLATS Ser909 were mainly detected in the nucleus at low cell density (Supplementary Figure S3B). However, the pYAP signals were scattered in whole cells at sparse cell density and localized in the cytoplasm only at high cell density (Supplementary Figure S3C). Moreover, the interactions of YAP with Par3, LATS1 and PP1A in the nucleus and in the cytoplasm were detected in co-immunoprecipitations with anti-YAP antibodies (Figure 4h); and similar interactions of LATS1 with Par3, YAP and PP1A were observed with anti-LATS1 antibodies (Supplementary Figure S3E). These data indicated that Par3 also forms a complex with YAP, recruiting PP1A and LATS1/2 in the nucleus, in addition to that in the cytosol.

### Dual function of Par3 in regulating YAP phosphorylation and activation

**Figure 5:**
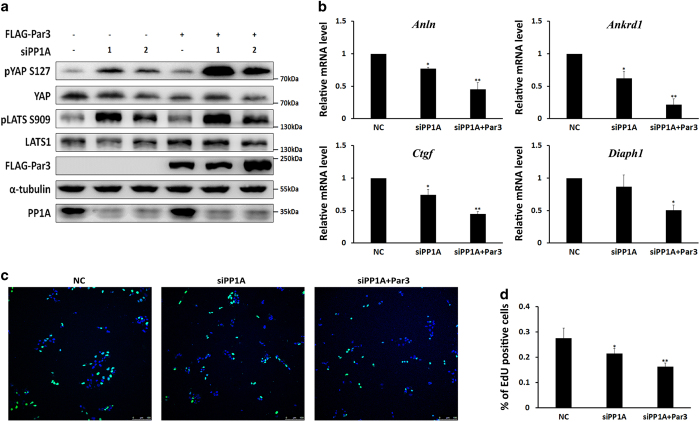
Dual function of Par3 in regulating YAP phosphorylation and activation**.** (**a**) PP1A knockdown alone increased YAP and LATS1 phosphorylation, and PP1A knockdown plus Par3 overexpression promoted more the increased phosphorylation of YAP and LATS1. 293T cells were transfected with two different siRNAs for PP1A and FLAG-Par3 plasmids. Western blot analysis was performed as indicated. (**b**) PP1A knockdown inhibited YAP target genes *Anln, Ankrd1, Ctgf* and *Diaph1*, and Par3 overexpression enhanced the inhibition of YAP target genes by PP1A knockdown. RT-qPCR was performed in 293T cells transfected with siRNA for PP1A and FLAG-Par3 at low cell density. (**c**) PP1A knockdown inhibited cell proliferation, and Par3 overexpression enhanced the inhibition of cell proliferation by PP1A knockdown, as assessed by an EdU assay. Total cell number was measured by 4′, 6-diamidino-2-phenylindole (blue), and cells in mitosis were labeled by EdU (green). Scale bar: 100 um. MDCK II cells were transfected with siRNA for PP1A and FLAG-Par3 plasmids for 2 days and were then cultured with normal medium containing 10 μM EdU for 30 min. Cells were then stained according to the Click-iT EdU Alexa Fluor 488 Imaging Kit (C10337) protocol from Invitrogen. (**d**) The ratio of EdU-positive cells/total cells was determined.

Functional outcomes of Par3 were regulated by its cell distribution and phosphorylation status, which are controlled by different cell signaling pathways. Thus, in particular, we sought to clarify the role of Par3 in regulating LATS1/2-YAP signaling in the absence of PP1A. As shown in Figure 5a, PP1A knockdown alone led to increased phosphorylation of both YAP and LATS1. Strikingly, Par3 overexpression in 293T cells, in which PP1A was knocked down, elevated robustly YAP hyperphosphorylation and also slightly increased LATS1/2 phosphorylation compared with that in cells with PP1A knockdown alone (Figure 5a). This observation suggested that increased Par3 may suppress YAP activity by promoting LATS1 activation and YAP phosphorylation in the case of PP1A suppression. Moreover, mRNA expression levels of YAP target genes, such as *Ankrd1, Anln, Ctgf* and *Diaph1*, were further inhibited in those cells by PP1A knockdown and Par3 overexpression (Figure 5b); cell proliferation was also inhibited, as assessed with 5-ethynyl-2'-deoxyuridine (EdU) assays (Figure 5c and d). Collectively, these data indicated that, contrary to its positive role in YAP activation, Par3 might actively suppress YAP activity, targeted genes and cell proliferation when PP1A is downregulated.

### The regulation of YAP by Par3 is a manner dependent on the cell context

**Figure 6:**
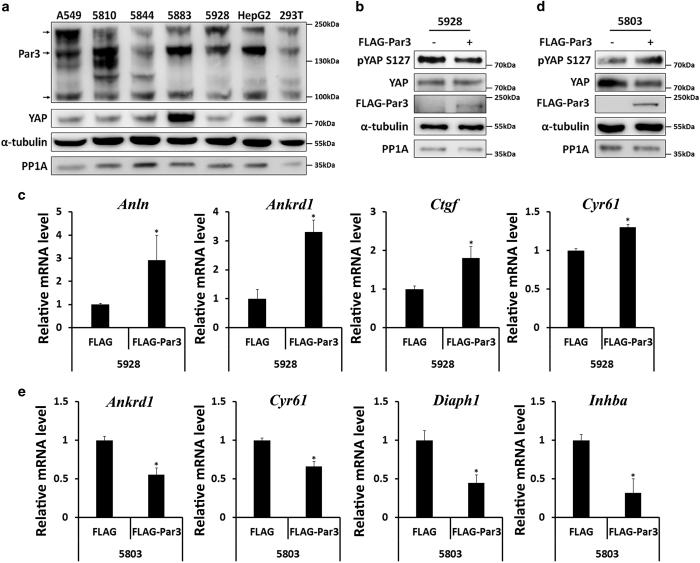
YAP activation by Par3 is differentially regulated in tumor cell lines**.** (**a**) Par3, YAP and PP1A expression patterns were different in different cell lines. The lung cell lines A549, 5810, 5844, 5883, 5928 and HepG2 were harvested for western blotting, and analyses were performed as indicated. (**b**) In the 5928 cell line, Par3 decreases YAP phosphorylation, as in 293T cells. 5928 cells were transfected with FLAG-Par3, and pYAP Ser127 was detected. (**c**) The YAP target genes *Anln, Ankrd1, Ctgf* and *Cyr61* were upregulated when Par3 was overexpressed. RT-qPCR was performed in 5928 cells transfected with FLAG-Par3 at low cell density. (**d**) In the 5803 cell line, Par3 increased YAP phosphorylation and reduced YAP protein levels. 5803 cells were transfected with FLAG-Par3, and pYAP Ser127 and YAP were detected. (**e**) The YAP target genes *Ankrd1, Cyr61, Diaph1* and *Inhba* were reduced when Par3 was overexpressed in the 5803 cell line. RT-qPCR was performed in 5803 cells transfected with FLAG-Par3 at low cell density.

The dual function of Par3 in cells (Figure 5) suggests that its biological function might be complex. Par3 overexpression has been found in different types of tumors [23, 41, 42]. Differential expression patterns of Par3, PP1A and YAP were detected in different tumor cell lines (Figure 6a). Thus, we asked whether Par3-regulated YAP phosphorylation is dependent on cell types or cell lines. YAP phosphorylation was investigated at low cell density in several lung cancer cell lines in which Par3 was transiently overexpressed. In the 5928 cell line, Par3 expression decreased YAP phosphorylation (Figure 6b), which was consistent with the results obtained from the 293T cells. However, in the 5803 cell line, Par3 expression increased YAP phosphorylation and reduced protein expression (Figure 6d), similarly to the results in 293T cells when PP1A was knocked down. Overexpression of Par3 led to upregulation and downregulation of YAP-targeted genes in 5928 cells (Figure 6c) and 5803 cells (Figure 6e), respectively. Together, these results showed that Par3 might exert different roles in regulating YAP phosphorylation and targeted gene expression in a physiological cell context- and/or cell type-dependent manner.

## Discussion

In this study, we illuminated novel signaling by a Par3–YAP complex that regulates the mammalian Hippo/YAP pathway in a manner dependent on cell density and cell–cell contact. The dynamic subcellular co-localization of Par3 and YAP was regulated by cell density. The Par3–YAP complex recruited PP1A and LATS1/2, promoting YAP hypophosphorylation and nuclear localization. Par3 activated YAP signaling, which mediated cell proliferation at low cell density but not at high cell density. In the case of PP1A depletion, Par3 promoted YAP hyperphosphorylation, which suppressed YAP activation and downstream targets. The dual function of Par3 in regulating YAP phosphorylation and activation was confirmed in different tumor cell lines.

The Hippo signaling pathway is highly conserved and limits organ size by phosphorylating and inhibiting the transcriptional co-activators YAP and TAZ in mammals and Yki in *Drosophila*, which are key regulators of proliferation and apoptosis [32]. More recent findings have implicated the Hippo-YAP pathway in cell–cell contact-mediated control of proliferation in cancer cells and normal developing tissues [24, 27, 43].

Par3, which contains multiple functional domains, including three PDZ domains, regulates TJ formation and positioning with respect to basolateral and apical membrane regions. In addition to the indispensable role in cell polarity, Par3 subcellular distribution has been implicated in mediating multiple cellular signaling pathways involving other cell functions [44, 45]. Our data indicated that Par3 co-localized with YAP at high cell density, primarily on the cell membrane; at low cell density, it primarily co-localized in the cytoplasm and the nucleus. Par3 knockdown had no obvious effect on YAP phosphorylation and activation at high cell density, but inhibited YAP activation and nuclear localization by increasing YAP phosphorylation at low cell density. Par3–YAP interaction is required for the regulation of YAP phosphorylation and nuclear localization. These findings suggested that Par3–YAP association has diverse roles in a physiological cell context or cell–cell contact manner. YAP interacts with several important polarity signal molecules, thereby coupling cell density and cell polarity with cell proliferation and apoptosis [26]. At high cell density, Par3, together with aPKC and Par6, forms the Par complex, which regulates TJ formation in the TJ region [5]. However, we did not detect any interaction of aPKC and Par6 with YAP (Supplementary Figure S2A), suggesting that Par3 regulates YAP signaling independently of the Par complex. A recent study has implicated that YAP has a dual function in the cell context-dependent transition from a proliferative to a differentiation state [46]. In developing lens epithelial cells, YAP may have a role in the proper organization of the polarized lens cells by interacting with the apical polarity complex including Crb complex. Interestingly, ASPP2 could also bind Par3 to be involved in TJ formation, and ASPP2 has been reported to bind to YAP via its WW domain in the TJ region [47]. Whether Par3 at TJs cooperates with YAP and/or ASPP2-YAP to maintain TJ formation and epithelial cell polarity needs to be further investigated.

YAP has been believed to be dephosphorylated in the cytoplasm and to translocate to the nucleus, where it binds to TEAD and induce gene expression, resulting in cell growth. Our data provided a hint that, at low cell density, Par3 may also affect YAP phosphorylation in the nucleus. This possibility is consistent with previous reports showing nuclear PP1 activity [40] and nuclear LATS1/2 localization in normal mesothelial Met-5A cells, human liver epithelial HepG2 cells and primary mouse fetal liver progenitor cells [39]. In MDCK cells, we also observed that Par3, LATS1/2 and PP1A interacted with each other in the nucleus (Figure 4 and Supplementary Figure S3), supporting the idea that the regulation of YAP phosphorylation by Par3 may occur in the nucleus.

Little is known about the role of Par3 in the nucleus. We have previously reported that γ-irradiation induces Par3 nuclear localization, thus demonstrating that Par3 is involved in double-strand break repair. HGF stimulation induces Par3 nuclear localization, thus suggesting a role for Par3 in HGF-induced endothelial–mesothelial transition in MDCK cells [15]. YAP dephosphorylation and nuclear localization induced by HGF has been reported by Kriegsheim’s group [48]. Interestingly, we did observe that Par3 knockdown in MDCK cells enhanced YAP phosphorylation induced by HGF (unpublished data), indicating a specific role of Par3 in regulating HGF-induced YAP activation. In addition, the PDZ-binding motif is necessary for YAP localization in the nucleus, the stabilization of p73 and the promotion of HEK293 cell apoptosis [49]. We showed that the third PDZ domain of Par3 directly interacts with the YAP PDZ-binding motif. Par3 knockdown notably diminished YAP shuttling between the nucleus and cytoplasm of MDCK cells in response to cell density and the Ca^2+^ on/off switch (Figure 1c and g and Supplementary Figure S1B). The Par3–YAP interaction was crucial for the regulation of YAP nuclear localization by Par3 (Figure 3e). Nevertheless, these data indicated that Par3 might have a direct role in regulating the YAP target gene regarding YAP-dependent cellular functions, besides regulating YAP phosphorylation and activity. In addition, whether Par3 directly interacts with YAP-TEAD complex in the nucleus to regulate the oncogenic activity of YAP is worth further investigating.

Currently, the mechanism by which Par3 regulates LATS and YAP phosphorylation remains elusive. LATS1/2 activation is required for YAP phosphorylation and inactivation [33] (Figure 4b and c). Par3 suppression led to increased LATS1/2 phosphorylation (Figure 4c). LATS1/2 knockdown notably diminished the increase in YAP phosphorylation caused by Par3 RNAi (Figure 4c). Thus, we suggest that Par3 may act primarily in transferring PP1A activity to inhibit LATS1/2 activation, thereby suppressing YAP phosphorylation. However, we could not exclude the possibility that the Par3-associated PP1A directly dephosphorylates YAP because with Par3 overexpression and LATS1/2 knockdown, a lower amount of YAP phosphorylation was observed compared with that in the context of LATS1/2 knockdown alone (data not shown). Similar results in a LATS1/2 knockout cell line have been reported previously [50]. LATS1 and YAP protein levels are reduced by Par3 overexpression when PP1A is knocked down (also see Supplementary Figure S4A for the dose-dependent effect of Par3 overexpression). This finding may be due to Par3-enhanced LATS1/YAP phosphorylation because phosphorylation of LATS1 and YAP has been reported to lead to their degradation [51, 52]. We cannot exclude the possibility that Par3 overexpression itself can decrease YAP protein levels. Notably, there was a decrease in total YAP signaling without enhanced YAP phosphorylation in H2170 cells (Supplementary Figure S4B). Par3 can bind to various important signal molecules, such as aPKC, LIMK2, Par1, PP1A and KIF3A, thereby mediating multiple cellular signaling pathways [53]. Traweger *et al*. [35] have shown that Par3 functions as a signaling platform, creating a focal point of enzyme activities in spatial cell compartments, coordinately recruiting aPKC, Par1 and PP1A, thereby affecting positive and negative phosphorylation events that regulate TJ formation. Par1 has been reported to regulate Yorkie activity by influencing Hippo phosphorylation status and Hippo-Salvador association [54]. Meanwhile, Par3 Ser144/973 phosphorylation by Par1 modulates Par3 activity and its cytoplasmic location [18]. Lv *et al*. have recently reported that Par1 phosphorylates PARD3 at S144/S873, thereby promoting PARD3 translocation from TJs to cytosol, where PARD3 promotes the interaction between LATS1 and PP1A, resulting in LATS1 dephosphorylation and inactivation. The Par3 Ser144/973 mutant, which primarily localizes to the membrane, is unable to decrease TAZ phosphorylation [50]. Both reduction and enhancement of YAP phosphorylation by Par3 have been identified in different lung cell lines (Figure 6b and d). These observations might suggest that the upregulation or downregulation of YAP phosphorylation and activity by Par3 is dependent on Par3’s spatial localization, phosphorylation state and cell context. This dependency may explain why Par3 has positive and negative effects on YAP phosphorylation in different lung cancer cell lines.

**Figure 7:**
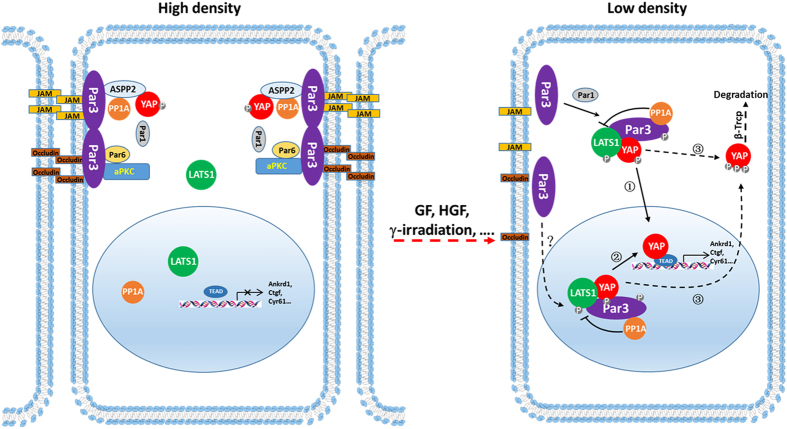
The hypothetical model for the dual function of Par3 in regulating YAP phosphorylation and activation. At high cell density, Par3 and YAP co-localize in the TJs at the cell–cell contact and Par3 has no effect on YAP phosphorylation (left panel). At low cell density, or calcium depletion, HGF stimulation/γ-irradiation, phosphorylated Par3 by Par1 translocates from membrane to the cytoplasm(①) or the nucleus (②). Cytoplasmic or nuclear Par3 recruits PP1A to dephosphorylate LATS1, promoting YAP activity (right panel). When PP1A is knocked down or Par3’s spatial localization is disordered, Par3 expression induces YAP hyperphosphorylation and degradation (③).

On the basis of the observations from this and previous works, it is plausible that Par3 has a dual role in regulating the Hippo-YAP signaling pathway by integrating cell density and/or cell polarity information in a cell-density and/or cell-context manner (Figure 7). At high cell density, Par3 mainly localizes at the TJs and forms the Par complex with Par6/aPKC for establishing/maintaining the TJ assembly and apical cell polarity. YAP colocalizes and interacts with Par3, ASPP2 and various other apical proteins [26] at the apical domain of the polarized cells, where YAP may contribute to conveying contact inhibition signals from the cell surface to the nucleus via Hippo pathway regulation [24, 25]. Alternatively, it may also have a role in maintaining cell polarity and cell shape via regulating correct localization of polarity proteins [46]. Excessive stimulations of extracellular cues such as calcium depletion, HGF, γ-irradiation are able to disrupt the adherens junctions, TJs and cell–cell contact, further leading to alteration of cell morphology and cell polarity. These alterations could promote cellular redistribution and co-localization of Par3 and YAP in the cytoplasmic and the nuclear, where Par3 forms a complex with YAP and LATS1 and recruits PP1A to dephosphorylate LATS1 and YAP, respectively, promoting YAP activity and downstream targets expression. Par3 phosphorylation by different kinases including Par1 [18, 50] may regulate the subcellular re-localization at low cell density. However, when PP1A is suppressed or Par3 overexpression possibly results in its mislocalization and/or over-activation in tumor cell lines, Par3 may bind to LATS1 and YAP without PP1A interaction, by which Par3 functions as a tumor suppressor to promote YAP phosphorylation and target gene repression, even YAP’s degradation. Thus, our results shed new light on a promising basis for the potential crosstalk between the Hippo pathway and cell polarity proteins in the regulation of cell proliferation.

## Materials and Methods

### Plasmids and siRNA

The human sourced Par3 long form (180 kDa form) plasmid was a gift provided by Dr Ian G Macara (University of Virginia, USA). The ΔPDZ1 domain (delete 251–385 aa), ΔPDZ2 domain (delete 425–549 aa) and ΔPDZ3 domain (delete 577–694 aa) of Par3 long form were amplified and inserted in-frame into pcDNA3.1-NFLAG by site-directed mutagenesis and PCR. Par3 PDZ1 domain (251–385 aa), Par3 PDZ2 (425–549 aa), Par3 PDZ3 (577–694 aa) were amplified and inserted in-frame into pGEX5T. The YAP plasmid and LATS1 plasmid were kindly provided by Dr Lei Zhang (SIBCB). The YAP/S127A plasmid was also generated by site-directed mutagenesis and PCR, and YAP/ ΔPDZM plasmid was generated as described by Oka and Sudol [49].

The sequences of the Par3 siRNA duplexes (siRNA1: GACAGACUGGUAGCAGUGU, siRNA2: CAUGGAGAUGGAGGAAUAC), LATS1/2 siRNA (LATS1 siRNA: CAUACGAGUCAAUCAGUAA, LATS2 siRNA: AAAGGCGUAUGGCGAGUAG) and PP1A siRNA (siRNA1: AAAACCTTCACTGACTGCTTC, siRNA2: CCATTCTTCTGGAGCTGGA) were synthesized by Genepharma (Shanghai, China).

### Antibodies

Anti-Par3 (07-330) was from Millipore (Darmstadt, Germany). Anti-YAP1 (WH0010413M1) for western blot, immunofluorescence and immunoprecipitation and anti-α-tubulin (T5168) were from Sigma (St Louis, MO, USA). Anti-phospho YAP (Ser127) (#4911), anti-LAT1 (#3477), anti-LATS2 (#5888) and anti-phosphoLATS (Ser909) (#9157) were from Cell Signaling Technology (Danvers, MA, USA). Anti-PP1A (A2184) was from AB clonal (College Park, MD, USA). Anti-Flag (M20008L) and anti-HA (M20003) were from Abmart (Shanghai, China). Lamin-B (sc-20682) was from Santa Cruz (Dallas, TX, USA).

### Cell culture and transfection

The HEK293T and MDCK cells were cultured in Dulbecco’s modified Eagle’s medium (Invitrogen, Waltham, MA, USA) supplemented with 10% calf serum at 37 °C and 5% CO_2_. The 293T cells were transiently transfected using Lipofectamine 2000 (Invitrogen), and MDCK cells were transfected with Lipofectamine LTX (Invitrogen). The siRNA transfections were performed using Lipofectamine RNAiMAX (Invitrogen).

### Calcium depletion and recovery experiment

MDCK II cells were grown to high density in Dulbecco’s modified Eagle’s medium-10% fetal bovine serum medium, which were washed twice with phosphate-buffered saline and incubated in a low Ca^2+^ medium containing 5% fetal calf serum, 3 mm Ca^2+^, penicillin and streptomycin for more than 20 h. For the Ca^2+^ recovery experiment, cells were cultured by replacing the low Ca^2+^ medium restored with 1.8 mm Ca^2+^ for the remainder of the time course.

### *In vitro* binding assay

Cells were lysed with 1 ml of lysis buffer (50 mm HEPES pH 7.5, 1% Triton X-100, 150 mm NaCl, 1 mm NaF, 100 μmol PMSF and Cocktail) and incubated with 3 μg purified glutathione-S-transferase fusion proteins conjugated to glutathione 4B beads. The beads were washed three times with HNTG buffer (20 mm HEPES pH 7.5, 150 mm NaCl, 0.1% Triton X-100, 10% glycerol) and eluted with sodium dodecyl sulfate sample buffer. Proteins were separated by sodium dodecyl sulfate-polyacrylamide gel electrophoresis and analyzed by Coomassie blue staining.

### Cell fractionation

This method was adapted from a previous protocol [55]. Briefly, the 293T and MDCK cells were supplemented with hypotonic buffer (10 mm Tris-Cl, 25 mm KCl, 10 mm NaCl, 1 mm MgCl_2_, 0.1 mm EDTA, 1 mm NaF (pH 7.2) and Cocktail) and were scraped off and passed through a 25- g needle 10 times, and centrifugated at 1000  r.p.m. for 10 min at 4 °C. The cytoplasmic fraction was taken from the supernatant. The resulting crude nuclear pellets were suspended with cell lysis buffer Ι (50 mm HEPES (pH 7.5), 10% glycerol, 0.5% Triton X-100, 150 mm NaCl and Cocktail) and centrifugated at 13 000  r.p.m. for 60 min at 4 °C. The final nuclear pellets were dissolved with RIPA buffer (50 mm HEPES (pH 7.5), 1% Triton X-100, 1% np-40, 0.1% sodium dodecyl sulfate, 150 mm NaCl, 1% deoxycholatic sodium, 1 mm NaF and Cocktail) and sonicated on ice.

### Immunoprecipitation and immunoblotting

Cells were lysed with 1 ml RIPA buffer and sonicated on ice. The lysates were quantified using the BCA Protein Assay Kit (TIANGEN Biotech, Beijing, China). The lysates were incubated with 4 ul antibody and 10 ul protein A/G beads (GE Healthcare, Marlborough, MA, USA) overnight at 4 °C, and the beads were washed three times with HTHG buffer and eluted with sodium dodecyl sulfate loading buffer. The proteins were separated and analyzed by western blot with the indicated antibodies.

### Immunofluorescence

MDCK cells were washed with cold phosphate-buffered saline three times, fixed with 2% PFA for 10 min and permeabilized with 0.5% Triton X-100 for 5 min. After blocking in 10% fetal bovine serum/(0.1%BSA/TBST) for above 30 min, cells were incubated with the first antibody diluted in 0.1% BSA/TBST overnight at 4 °C. After washing with 0.1%BSA/TBST for three times, cells were incubated with Alexa Fluor 488-, 546- or 647-conjugated secondary antibodies (1:1000 dilution, Invitrogen) and 4’, 6-diamidino-2-phenylindole (Sigma) for 1 h at room temperature. Then, cells were subjected to laser-scanning confocal microscopy (Leica, TCS SP5, Wetzlar, Germany).

### Quantitative real-time PCR

MDCK cells were harvested in TRIzol (Invitrogen) for total RNA extraction, and RNA was reverse-transcribed with a PrimeScript RT reagent kit with a gDNA Eraser (TaKaRa, Kusatsu, Japan). Quantitative real-time PCR (qRT-PCR) analyses were performed with SYBR Green Master (ROX; Roche, Indianapolis, IN, USA) in an ABI 7500 Fast thermal cycler and were analyzed with 7500 Software v2.0.1. All expression levels are reported relative to glyceraldehyde-3-phosphate dehydrogenase (GAPDH). PCR oligo sequences are listed as follows:

Par3

For: GCAGTGACCCGGCTTTAATT; Rev: CAGCAGTGTTTTGCTTCAG

Ankrd1

For: AGCCCAGATCGAATTCCGCG; Rev: CTCCTTCTCGGTCTTTGGCAT

Anln

For: ATGTCTTCGTGGCCGATTTGA; Rev: CTCTGACAGTGAGTTTCCTGTTT

Cyr61

For: TGAAGCGCCTCCCAGTTTT; Rev: CGGGTCTCCTTCACCAGGCG

Ctgf

For: ACCGACTGGAAGACACGTTTG; Rev: CCAGGTCAGCTTCACAAGG

Diaph1

For: GCGGTATGCATTGTAGGGGA; Rev: CAGGAGATGTAACCAGGGCA

Inhba

For: GCTGCACGTAGGCAAAGTCG; Rev: GCTGTGCCTGATTCCGCGAA

GAPDH

For: ACGGATTTGGCCGTATTGGGC; Rev: TTGACTGTGCCGTGGAATTTG

### Proliferation assay

To determine the cell proliferation rate, we used a traditional MTT assay (Sigma). Cells were seeded in a 96-well microplate at a concentration of 2000 cells/well after transfection with siRNA and plasmids. The medium was changed every day. Survival was evaluated by replacing the culture medium with 100 μl of 1 mg ml^−1^ MTT-media solution every day. This treatment was followed by 4 h of incubation at 37 °C. Then, the MTT reagent was removed before addition of 100 μl of dimethyl sulfoxide. The absorbance was determined at 570 nm with a microplate reader.

### EdU assay

The EdU assay was performed with a Click-iT EdU Alexa Fluor 488 Imaging Kit (C10337) from Invitrogen, which is an alternative to the 5-bromo-2’-deoxyuridine assay. A total of 2×10^5^ MDCK II cells/well were seeded in a 24-well plate and were transfected with PP1A siRNA and Flag-Par3 plasmids. After 48 h, 1×10^5^ cells/well were seeded in a new 24-well plate with coverslips for an EdU assay. Cells were treated with 10 μM EdU in Dulbecco’s modified Eagle’s medium. The procedures were performed exactly according to the manufacturer’s instructions.

### Image analysis

We used a Columbus Image Data Storage and Analysis System to analyze confocal pictures. First, we identified the nucleus (dyed with 4′,6-diamidino-2-phenylindole, blue); then, we measured the entire fluorescence intensity of YAP and the fluorescence intensity of the portion merged with the nucleus, which represented nuclear YAP; the difference in intensity between the entire YAP signal and the nuclear YAP signal represented the cytoplasmic YAP signal. At each time point, we analyzed at least three images, each containing 120~150 cells/picture (at low cell density, the cell number was less than 50 cells/picture).
