# Supplementary material for: Dual function of partitioning-defective 3 in the regulation of YAP phosphorylation and activation
Source: Cell Discov. 2016 Jul 5;2:16021–. doi: 10.1038/celldisc.2016.21 (PMC4932730; doi:10.1038/celldisc.2016.21)
Supplement: Supplementary Figure S1 [file celldisc201621-s1.pdf]

Supplemental figures

Figure S1:

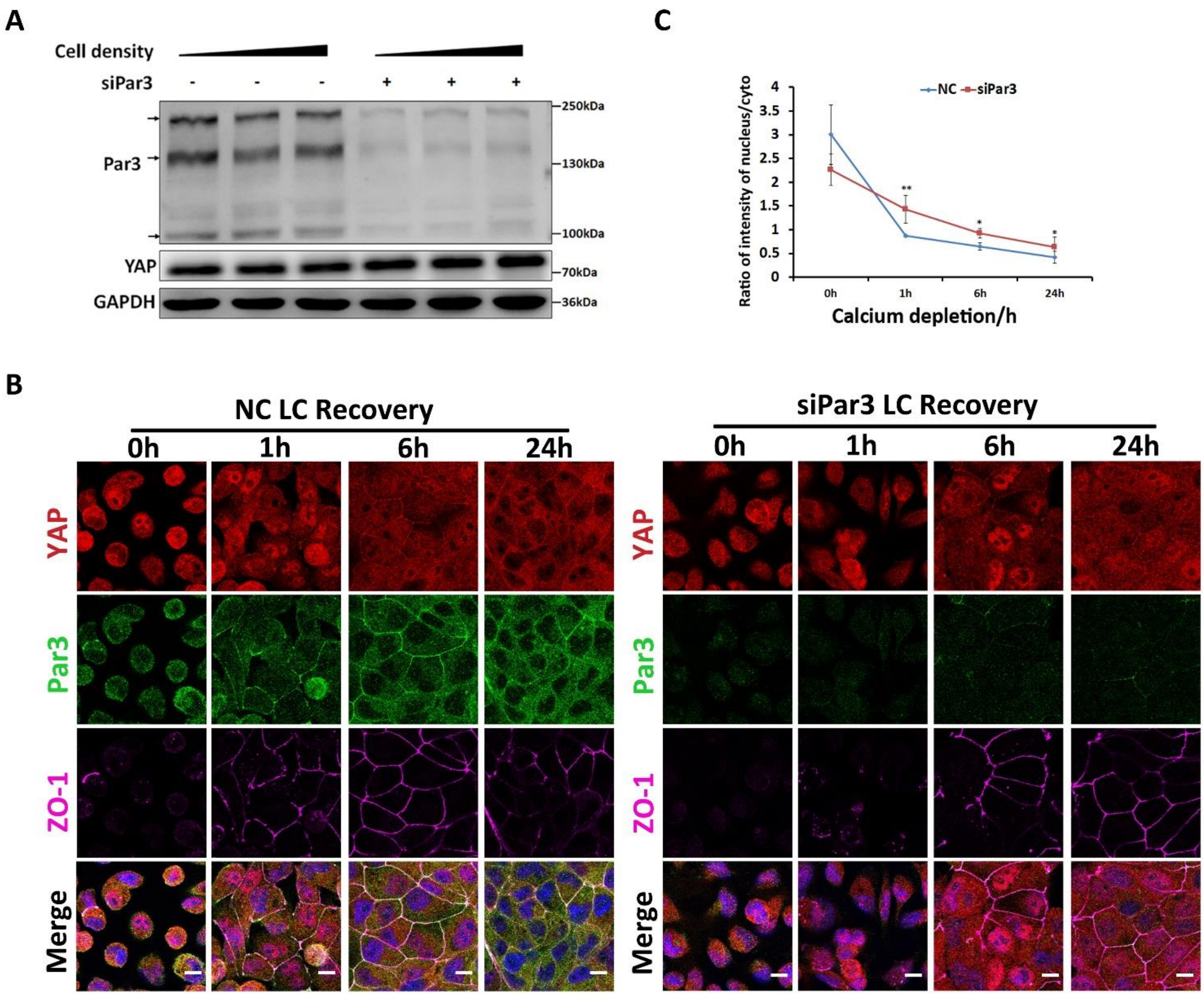

Figure S1. Par3 knockdown reduced YAP translocation to cytoplasm in Ca^{2+} switch on system

(A) The western blot showed that the efficiency of Par3 knockdown and YAP protein level at different cell density in MDCKⅡ cells.

(B) Par3 knockdown reduced YAP translocation in Ca^{2+} switch on system. Representative images of YAP translocation into the cytoplasm at high density when Ca^{2+} was depleted for 24h first and Ca^{2+} recovery in the time course are shown. After 1days while MDCKⅡ cells were transfected with siRNA for Par3 , MDCKⅡ were cultured with Ca^{2+} free media for 24h and with normal media in the indicated time course. YAP (red), Par3 (green) and ZO-1 (purple). Scale bar: 25um.

(C) Ratios of the percentages of nuclear and cytoplasmic YAP and Par3 at different time points. Pictures were analyzed by Columbus Image Data Storage and Analysis System. **p<0.01. *p<0.05.
