# Supplementary material for: Dual function of partitioning-defective 3 in the regulation of YAP phosphorylation and activation
Source: Cell Discov. 2016 Jul 5;2:16021–. doi: 10.1038/celldisc.2016.21 (PMC4932730; doi:10.1038/celldisc.2016.21)
Supplement: Supplementary Figure S2 [file celldisc201621-s2.pdf]

Figure S2

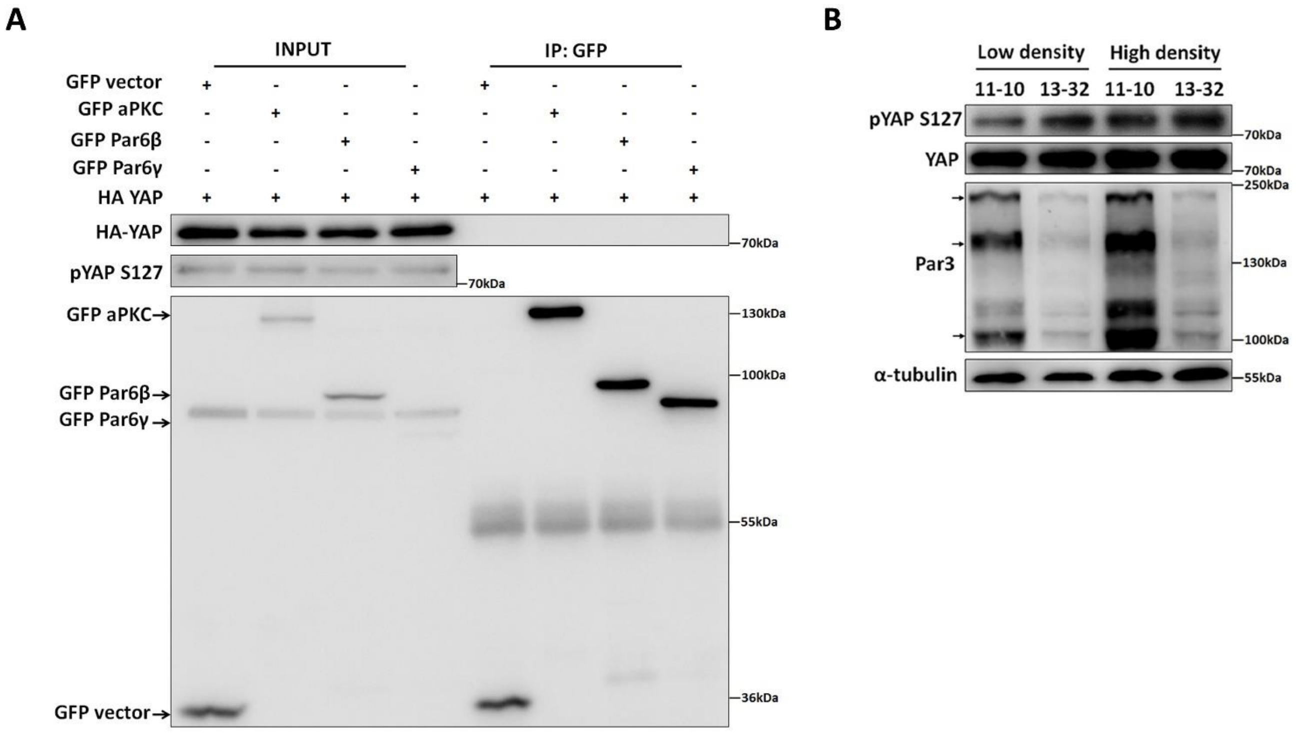

Figure S2. YAP regulation by Par3 is independent of aPKC and Par6

(A) aPKC and Par6 couldn't interact with YAP and regulate YAP phosphorylation. Co-immunoprecipitation of GFP-aPKC, GFP-Par6β, GFP-Par6γ and HA-YAP was performed with GFP antibody in 293T cells. pYAP Ser127 site was also detected.

(B) In Par3 knockdown stable MDCK Ⅱ cell lines, Par3 knockdown increased YAP phosphorylation at low cell density but not at high density. 11-10 was the negative control cell line; 13-32 was the Par3 knockdown cell line.
