# Supplementary material for: Dual function of partitioning-defective 3 in the regulation of YAP phosphorylation and activation
Source: Cell Discov. 2016 Jul 5;2:16021–. doi: 10.1038/celldisc.2016.21 (PMC4932730; doi:10.1038/celldisc.2016.21)
Supplement: Supplementary Figure S3 [file celldisc201621-s3.pdf]

Figure S3:

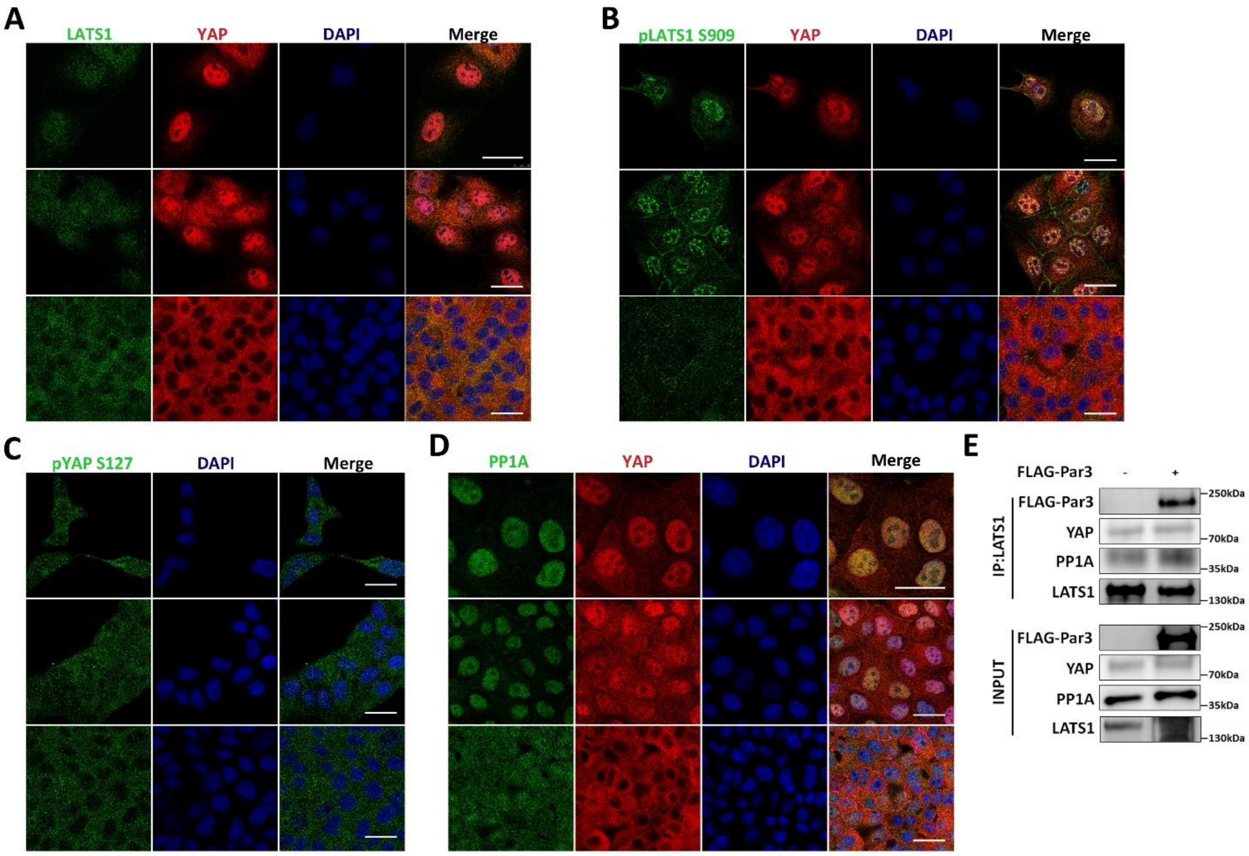

Figure S3. Par3 may regulate the phosphorylations of YAP and LATS1 in the nucleus at low density

(A-D) The localization of LATS1(green in Fig S3A), pLATS S909(green in Fig S3B), pYAP S127(green in Fig S3C), PP1A(green in Fig S3D) and YAP(red) in MDCKⅡ cells at different cell density. The endogenous YAP, LATS1, pLATS Ser909, PP1A and pYAP Ser127 were detected in MDCKⅡ cells at different cell density; scale bar 25um.

(E) LATS1 interacts with FLAG-Par3, YAP and PP1A. IP with anti-YAP antibodies was conducted in 293T cells at low cell density. Western blot analysis was performed as indicated.
