# Supplementary material for: Dual function of partitioning-defective 3 in the regulation of YAP phosphorylation and activation
Source: Cell Discov. 2016 Jul 5;2:16021–. doi: 10.1038/celldisc.2016.21 (PMC4932730; doi:10.1038/celldisc.2016.21)
Supplement: Supplementary Figure S4 [file celldisc201621-s4.pdf]

Figure S4:

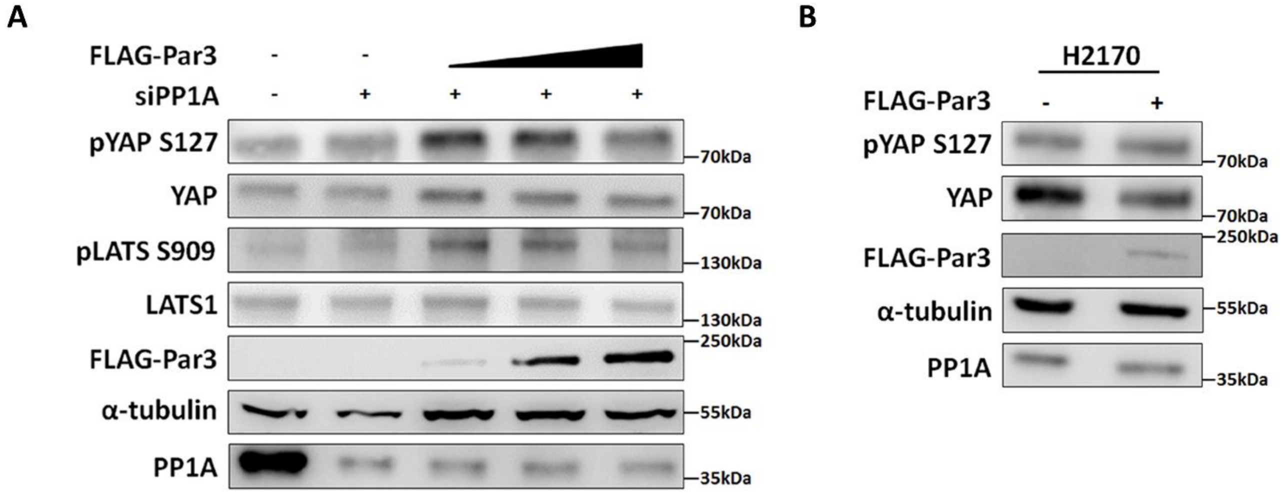

Figure S4. Par3 expression may lead to YAP degradation.

(A) Overexpression of Par3 induced YAP degradation when PP1A was knocked down. 293T cell were transfected with siRNA for PP1A and dose dependent FLAG-Par3 plasmid; western blot analysis was performed as indicated. (B) Par3 decreased YAP protein level in H2170 cell. H2170 cells were transfected with FLAG-Par3 and pYAP Ser127 and YAP were detected.
